# Role of Na^+^-K^+^ ATPase enzyme in vascular response of goat ruminal artery

**DOI:** 10.4103/0253-7613.51343

**Published:** 2009-04

**Authors:** K. Kathirvel, S.C. Parija

**Affiliations:** Department of Pharmacology and Toxicology, Faculty of Veterinary Sciences and Animal Husbandry, Orissa University of Agriculture and Technology, Bhubaneswar - 751 003, India

**Keywords:** Barium, K^+^ channel, K^+^- ATPase, Na^+^, oubain, ruminal artery

## Abstract

**Objective:**

To study the role of Na^+^, K^+^- ATPase enzyme in the vascular response of goat ruminal artery.

**Materials and Methods:**

Ruminal artery was obtained in chilled aerated modified Krebs-Henseleit solution (KHS) from a local slaughterhouse and transported in ice for further processing. The endothelium intact arterial ring was mounted in a thermostatically controlled (37 ± 0.5°C) organ bath containing 20 ml of modified KHS (pH 7.4) bubbled with oxygen (95%) and CO_2_ (5%) under 2g tension. An equilibration of 90 min was allowed before addition of drugs into the bath. The responses were recorded isometrically in an automatic organ bath connected to PowerLab data acquisition system. In order to examine intact functional endothelium, ACh (10 *μ*M) was added on the 5-HT (1.0 *μ*M) - induced sustained contractile response. Similarly, functional characterization of Na^+^, K^+^-ATPase activity was done by K^+^-induced relaxation (10 *μ*M-10 mM) in the absence and presence of ouabain (0.1 μM/ 0.1 mM), digoxin (0.1 μM) and barium (30 μM).

**Results:**

ACh (10^−5^ M) did not produce any relaxing effect on 5-HT-induced sustained contractile response suggesting that vascular endothelium has no significant influence on the activation of sodium pump by extracellular K^+^ in ruminal artery. Low concentration of Ba^2+^ (30 μM) (IC_50_: 0.479 mM) inhibited K^+^-induced relaxation suggesting K_ir_ (inward rectifier) channel in part had role in K^+^-induced vasodilatation in ruminal artery. Vasorelaxant effect of KCl (10 μM-10 mM) in K^+^-free medium is also blocked by ouabain (0.1 μM and 0.1 mM) (IC_50_:0.398 mM and IC_35_: 1.36 mM), but not by digoxin (0.1 μM) (IC_50_ 0.234 mM) suggesting that ouabain sensitive Na^+^, K^+^-ATPase isoform is present in the ruminal artery.

**Conclusion:**

In the goat ruminal artery functional regulation of sodium pump is partly mediated by K^+^ channel and ouabain sensitive Na^+^, K^+^ ATPase.

## Introduction

Na^+^,K^+^-ATPase is an enzyme of the plasma membrane of most cells that uses cellular ATP to exchange cytoplasmic Na^+^ for extracellular K^+^.[1] The function of the Na,K-ATPase is essential for the generation and maintenance of the electrochemical gradients.[2] Structurally, Na^+^,K^+^-ATPase is an oligomer that is composed of distinct molecular forms of two major polypeptides, the α and β subunits.[3] At present, four structural variants of the α polypeptide (α_1_, α_2_, α_3_ and α_4_) and three β (β_1_, β_2_ and β_3_) subunits have been identified in mammals. Association of the α and β polypeptides in different oligomers results in multiple isozymes of the Na^+^,K^+^-ATPase that have unique functional properties and a tissue-specific pattern of expression.[3]

The sodium pump, in turn, is the target for multiple regulatory mechanisms.[4] It is also responsible for maintaining tone and contractility of smooth muscle.[5] Ouabain, a cardiotonic steroid, has been shown to be an endogenous factor that is secreted by the adrenal glands in humans and other mammals and is present in blood at nanomolar concentrations.[6] The mechanism of action of ouabain has been attributed classically to ion changes that are secondary to inhibition of the catalytic and transport activity of the Na^+^,K^+^-ATPase.[7] Four different isoforms, namely α_1_, α_2_, α_3_ and α_4_ with different ouabain affinities are expressed in different species and in a tissue-specific manner.[8] Regional variations in activity and isoform-specific expression of sodium pump in vascular tissues have been reported.[9] The presence of a microsomal Na^+^,K^+^-ATPase in sheep rumen epithelium activity which is reduced by 50% in the presence of ouabain has been reported.[10] Studies using biopsies of rumen epithelium papillae measured a net influx of [^86^Rb] across the canine ruminal epithelium[11] and these findings are similar to a high concentration of Na^+^, K^+^-ATPase found in [^3^H] ouabain-binding studies.[12] But there is no research related to ruminal artery to date on Na^+^, K^+^-ATPase and its role in maintaining the vascular tone in ruminal artery. Therefore, the present study was undertaken to identify the sensitivity of Na^+^, K^+^-ATPase to ouabain in goat ruminal artery.

## Materials and Methods

### Preparation of ruminal arterial ring and tension recording

After careful exposure of the goat celiac artery, the branch of the right ruminal artery was dissected out and placed in a cold aerated modified Krebs-Henseleit saline (MKHS) solution. Arteries were cleared of fat and connective tissue and cut into rings of about 2-2.5 mm in length. The arterial ring was then mounted between two stainless steel L-shaped hooks made of 28 gauge stainless steel wire and kept under resting tension of 2 g in a thermostatically controlled (37.0 ± 0.5°C) automatic organ bath (Pan Lab) of 20 ml capacity, containing MKHS and was aerated continuously with air. The arterial rings were equilibrated for 1.5 h before recording the muscle tension. During this period, the bathing fluid was changed every 15 min. The change in tension was measured by a highly sensitive isometric force transducer (Model: MLT 0201, AD instruments, Australia) and recorded in a PC using chart 5.0 Pro software.

### ACh (10^-6^ M) on 5-HT- induced sustained contractile response

In order to examine intact functional endothelium, a single sub-threshold dose of 5-HT (1 *μ*M) was added to the bath after equilibration. ACh (10 *μ*M) was added to the bath with contact period of 1 min as soon as 5-HT induced contractile response was maintained at plateau. The effect of ACh (10 *μ*M) on the 5-HT (1 *μ*M) - induced sustained contractile response was examined considering the 5 HT- induced contraction at plateau as 100%. This procedure was followed in each experiment to study Na^+^,K^+^-ATPase activity. The functional activity of Na^+^,K^+^-ATPase was indirectly measured using the method described by Webb and Bhor.[13] In brief, in the absence of ACh effect on 5-HT induced sustained contraction, the bath solution was replaced by modified KHS and arterial ring was incubated for 30 min and solution changed at 15 min intervals for further experiments.

### Experiments with K+-free physiological solution

K^+^-induced relaxation of arterial segments exposed to K^+^-free MKHS is an experimental protocol for functional assessment of vascular sodium pump. In order to study the regulation of the ruminal artery sodium pump by vascular endothelium and different protein kinases, tissues were equilibrated in MKHS for 90 min and then the tissue viability was checked with 5-HT (1.0 *μ*M). The endothelial integrity was examined by applying ACh (10 *μ*M) to the vessels pre-constricted with 5-HT (1 *μ*M). Then, after a period of wash for 30 min in MKHS, tissues were incubated in K-free solution and all the subsequent experimental protocols were carried out in K^+^- free solution. Incubation of ruminal arterial segment in K^+^-free solution generated a rise in basal tension which was not stable (and was noted in 2-3 experiments). In order to obtain a stable contraction in K^+^- free medium submicromolar concentration of 5-HT was added to K^+^- free PSS before the subsequent experimental protocol.

### KCl-induced relaxation in K^+^- free MKHS in presence of barium

**Figure 1:**
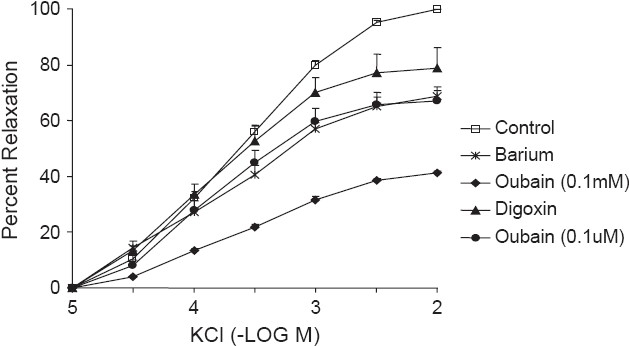
Effect of the digoxin (0.1 μM), Ba^++^(30 μM) and ouabain (0.1 μM, 0.1 mM) on KCl (10 μM-10 mM) induced relaxation in goat ruminal artery pre-contracted with K^+^-free MKHS. The results represent the mean ± SEM of the control

In another set of experiments, the tissue was incubated in K^+^- free MKHS containing barium (30 *μ*M) for 30 min to obtain a sustained contraction and KCl was added (10 *μ*M -10 mM) to the bath cumulatively at an interval of 1 min and relaxation was recorded [Figure 1].

### KCl-induced relaxation in K^+^- free MKHS in presence of digoxin

Ruminal artery was incubated with digoxin (0.1 *μ*M) for 30 min to obtain a sustained contraction in K^+^-free MKHS and KCl (10*μ*M -10 mM) was added to the bath cumulatively with intervals of 1 min between two adjacent concentrations of KCl [Figure 1].

### KCl-induced relaxation in K^+^- free MKHS in presence of ouabain

Similarly, ruminal artery was incubated with ouabain (0.1 *μ*M /0.1 *μ*M) for 30 min in K^+^-free MKHS to obtain a sustained contraction. KCl (10 *µ*M -10 mM) was added to the bath cumulatively with intervals of 1 min between adjacent concentrations and relaxation was recorded [Figure 1].

### Data analysis

The results were analyzed by interactive non-linear regression through the computer program GraphPad Prism (GraphPad Prism Software, San Diego, CA, USA).

### Drugs

Digoxin (Samrath Pharma, India), acetylcholine chloride, ouabain and serotonin (Sigma Aldrich, USA) were employed in this study.

## Results

### Effect of ACh on 5-HT (0.1 *μ*M) induced contraction and KCl on K+-free medium

Pre-constriction of the ruminal artery ring was achieved by 5-HT (1 *μ*M) following equilibration for a period of 90 min. 5-HT contracted the arterial ring reaching a steady level (1.25 ± 0.24 g; n = 6) within 6 to 8 min. Additions of ACh did not induce relaxation suggesting lack of function of endothelium. Following washes with MKHS, the basal tone was restored. Exposure of arterial ring to K^+^-free solution caused a rise in basal tone that reached the maximum (0.86 ± 0.05 g n = 6) in about 20-25 min. The K^+^-free contracture was not steady and often slowly decayed over a period of time. It was, therefore, difficult to plot concentration response curve to KCl (10 *μ*M to 10 mM). Re-exposure of the same arterial rings to the same K^+^-free solution showed a decrease in baseline tension as compared to initial exposure. Addition of 5-HT, 0.1 *μ*M to K^+^-free medium caused a steady contracture. KCl (10 *μ*M to 10 mM) added cumulatively at half log increments caused concentration-dependent relaxation with a pD'_2_ of 3.65 ± 0.04 (n = 32) [Table 1 and Figure 1].

**Table 1:** Effect of digoxin, barium and ouabain on KCl induced vasorelaxation in goat ruminal artery

*Treatment*	*n*	*E_max_/EB_max_ (%)*	*PD'_2_*	*IC_50_ [95% CL] (M)*
Nil (KCl control)	32	100	-	2.22 [1.99 − 2.43] × 10^−4^
Digoxin (0.1 μM)	5	79.9 ± 1.22***	7.75 ± 0.12	2.34 [1.78 − 3.1] × 10^−4^
Ba^++^ (30 μM)	6	68.4 ± 2.65***	5.01 ± 0.2	4.79 [3.78 − 6.05] × 10^−4^***
Ouabain (0.1 μM)	6	68.8 ± 0.7***	7.45 ± 0.1	3.98 [3.31 − 4.79] × 10^−4^***
Ouabain (0.1 mM)	6	41.5 ± 1.12	5.4 ± 0.2	1.36 [0.89 − 2.14] × 10^−3^ (at IC_35_)

Values are mean ± SEM;

*** *P* < 0.001 when compared with control

### Effect of digoxin, barium and ouabain on KCl-induced relaxation

Exposure of arterial ring to K^+^-free solution following equilibration with physiological solution and of tissue viability testing with 5-HT (1.0 *μ*M) showed a slow rise in baseline tension with maximum value of 0.62 ± 0.09 g (n = 6) over a period of 20-25 min. When 5-HT (0.1 *μ*M) was added to the declining phase of K^+^-free contracture, the steady state was achieved (mean contracture, 0.86 *μ* 0.04 g, n = 32) and then the relaxation response curve of KCl was elicited in presence of Ba^2+^ or digoxin or ouabain.

The mean maximal inhibition (I_Bmax_) of KCl-induced relaxation response curve was significantly (*P* < 0.001) by Ba^2+^ (31.7 ± 2.7%), digoxin (20.1 ± 1.2%), ouabain at 0.1 *μ*M (31.2 ± 0.7%) and 0.1 mM (58.5 ± 1.2%) as compared to pooled control (100%). The KCl -induced relaxation response curve was shifted to right in presence of Ba^2+^, ouabain at 0.1*μ*M and 0.1 mM with significant decrease in the mean IC_50_. In the presence of ouabain (0.1 mM), there was clear-cut rightward shift of the KCl-induced inhibitory response curve without attainment of IC_50_. As in the presence of ouabain (0.1 mM) the maximal inhibition was less than the 50% (E_Bmax_: 41.5 ± 1.12%), the IC_35_ was calculated for comparison. The IC_35_ of the KCl-induced inhibitory response curve in presence of ouabain (0.1 mM) (IC_35_:1.36 mM) was about 10.23 fold more than the control (IC_35_: 0.139 mM). The mean I_Bmax_, pD'_2_ and IC_50_ of the KCl-induced relaxation response curve in presence of Ba^2+^ or digoxin or ouabain were compared with the control in the Table 1 and curves are presented in Figure 1.

## Discussion

Na^+^-K^+^-ATPase exists in the plasma membrane as a heterodimer consisting of a catalytic α-subunit and a glycosylated β-subunit.[14] In vascular smooth muscle, the occurrence of α_1,_ α_2_ and α_3_ subunits has been reported in rat mesenteric artery,[9] rat aorta myocytes,[15] rat thoracic, superior mesenteric and tail arteries.[16] However, the role of sodium pump has not been characterized in the ruminal artery of the ruminant species. Similarly, there is limited information with respect to regulation of the ruminal arterial sodium pump by agonist. Using ruminal artery as a model for ruminant vascular smooth muscles, we employed isometric tension recording to study KCl-induced relaxation of goat ruminal artery rings contracted with K^+^-free solution and low concentration of 5-HT to study the functional regulation of sodium pump. There are several findings of the present study. First, endothelium of ruminal artery had no significant influence on the activation of sodium pump by extracellular K^+^ and small increases in the extracellular concentration of K^+^ (of < 30 mM) caused concentration related relaxation of the goat ruminal artery in K^+^ free medium, confirming that K^+^ powerfully relaxes this artery. Second, this response to K^+^ was significantly inhibited by 30 mM Ba^2+^, which, at this concentration, is considered a selective inhibitor of the inward rectifier K^+^ (K_ir_) channel.[17,18] By contrast, ouabain had a significant inhibitory effect on K^+^-induced vasorelaxation in a concentration dependent manner, suggesting that the Na^+^-K^+^-ATPase has a potential role in ruminal vasodilatation. Third, digoxin at sub micromlar concentration (0.1 *μ*M) significantly inhibited the K^+^- induced relaxation at only 3 and 10 mM. These observations clearly demonstrated the involvement of K_ir_ channel and Na^+^-K^+^-ATPase in K^+^ induced vasorelaxation of the goat ruminal artery.

KCl-induced relaxation in vascular smooth muscles may involve several independent mechanisms, such as activation of sarcolemmal Na^+^-K^+^-ATPase and/or activation of inward rectifier K^+^-channels.[9] K^+^-induced dilation of small renal artery was attributed to the activation of smooth muscle Na^+^-K^+^-ATPase with no role for inward rectifier K^+^-channel.[19] One of the distinguishing features of vascular relaxation by K^+^ involving Na^+^- K^+^-ATPase is that the extracellular concentration of K^+^ is less than 5 mM, whereas inward rectifier K^+^-channels primarily mediate K^+^-induced relaxation above the physiological K^+^-concentration (>5 mM).[18] In goat ruminal arteries, we found that extracellular K^+^ between 10 *μ*M and 10 mM produced graded relaxation of the vessels bathed in K^+^-free solution and primed with 5-HT (0.1 *μ*M) to sustain the contractions.

Activation of the K_ir_ channel and conduction of an outward K^+^ current in response to small increases in extracellular K^+^ is thought to occur because of unique gating properties of K_ir_ channels.[17,18] Larger increases in K^+^ (by < 30 mM) cause smooth muscle depolarization and subsequent constriction of several arteries due to marked membrane depolarization and Ca^2+^ entry via voltage-operated Ca^2+^ channels.[18] Recent findings suggest that the K_ir_ channel is indeed involved in mediating vascular smooth muscle hyperpolarization and vasorelaxation in response to K^+^.[19,20] The present study provided evidence that K^+^- induced vasorelaxation in ruminal artery is Ba^2+^ sensitive, and is likely to be at least partly mediated by activation of K_ir_ channels. Ba^2+^ (30 mM) can selectively abolish smooth muscle relaxation in response to K^+^, whereas selective inhibitors of other K^+^ channels do not inhibit K^+^-induced relaxation,[20] suggesting that these concentrations of Ba^2+^ are sufficient to completely inhibit K_ir_ channels and that K^+^ is likely to activate only this type of K^+^ channel. Interestingly, we observed only partial (31.7 ± 2.7%) inhibition of K^+^- induced vasorelaxation by Ba^2+^ in this study, which is in contrast to findings of the studies in which Ba^2+^ was reported to abolish completely the K^+^-induced vasorelaxation in blood vessels.[20] The discrepancy in the sensitivity to Ba^2+^ of the K^+^-induced vasorelaxation versus hyperpolarization could be related to either altered vascular response specific to ruminal artery or an influence of several mediators that participate in ruminal contraction.

When the ruminal arteries were immersed in a K^+^-free medium a small and transient contraction appeared. In other vessels, it has been reported that this response ranges from no increase in tension to a marked contraction[21,22] suggesting different Na^+^ pump activities in the vascular beds. These contractions are due to Na^+^ pump inhibition produced by K^+^-omission.[23,24] The subsequent addition of K^+^ elicited vasodilatation which was blocked by ouabain more potently than digoxin. The inhibitory effect on K^+^-induced vasorelaxation was further blocked in presence of higher concentration (0.1 *μ*M) of ouabain. This finding clearly demonstrates that ouabain inhibits Na^+^ pump in the ruminal artery in a concentration-related manner.

Consistent with the expression of the sodium pump isoforms in rodents, recent studies on gene targeted mice emphasize a significant role of the α_2_ isoform in regulating contractility of blood vessels *in vitro* and regulation of blood pressure *in vivo.*[25,26] The α_1_ isoform has been found to be having a “housekeeping role” in mouse aorta[26] and α_2_ isoform has been shown to possess a high affinity to low (submicromolar) concentrations of ouabain[27] in pulmonary vasculature. Thus far there is no information on the physiological roles of α_1_ and α_2_ isoforms in goat ruminal artery; the present functional study suggests that this isoform may have a role in regulating contractility of the ruminal vasculature

In conclusion, the results of this study demonstrate that K^+^ elicits marked relaxation of the goat ruminal artery. This effect may be (i) in part mediated through Ba^2+^ sensitive K_ir_ channel, as well as (ii) ouabain sensitive Na,K-ATPase isoform which may contribute to the vasorelaxant response. Further study is needed to confirm the molecular identity of Na,K-ATPase in goat ruminal artery.
